# Metformin Decreases Reactive Oxygen Species, Enhances Osteogenic Properties of Adipose-Derived Multipotent Mesenchymal Stem Cells* In Vitro*, and Increases Bone Density* In Vivo*

**DOI:** 10.1155/2016/9785890

**Published:** 2016-04-18

**Authors:** Krzysztof Marycz, Krzysztof A. Tomaszewski, Katarzyna Kornicka, Brandon Michael Henry, Sebastian Wroński, Jacek Tarasiuk, Monika Maredziak

**Affiliations:** ^1^Electron Microscopy Laboratory, Wroclaw University of Environmental and Life Sciences, 5b Kozuchowska Street, 51-631 Wroclaw, Poland; ^2^Wroclaw Research Centre EIT+, 147 Stablowicka Street, 54-066 Wroclaw, Poland; ^3^Department of Anatomy, Jagiellonian University Medical College, 12 Kopernika Street, 31-034 Krakow, Poland; ^4^Faculty of Physics and Applied Computer Science, AGH University of Science and Technology, 30 Mickiewicza Street, 30059 Krakow, Poland; ^5^Department of Animal Physiology and Biostructure, Faculty of Veterinary Medicine, Wroclaw University of Environmental and Life Sciences, 31 Norwida Street, 50-375 Wroclaw, Poland

## Abstract

Due to its pleiotropic effects, the commonly used drug metformin has gained renewed interest among medical researchers. While metformin is mainly used for the treatment of diabetes, recent studies suggest that it may have further application in anticancer and antiaging therapies. In this study, we investigated the proliferative potential, accumulation of oxidative stress factors, and osteogenic and adipogenic differentiation potential of mouse adipose-derived stem cells (MuASCs) isolated from mice treated with metformin for 8 weeks. Moreover, we investigated the influence of metformin supplementation on mice bone density and bone element composition. The ASCs isolated from mice who were treated with metformin for 8 weeks showed highest proliferative potential, generated a robust net of cytoskeletal projections, had reduced expression of markers associated with cellular senescence, and decreased amount of reactive oxygen species in comparison to control group. Furthermore, we demonstrated that these cells possessed greatest osteogenic differentiation potential, while their adipogenic differentiation ability was reduced. We also demonstrated that metformin supplementation increases bone density* in vivo*. Our result stands as a valuable source of data regarding the* in vivo* influence of metformin on ASCs and bone density and supports a role for metformin in regenerative medicine.

## 1. Introduction

Over the past several years, the pleiotropic effects of metformin (N, N′-dimethylbiguanide), a commonly used drug for the treatment of type 2 diabetes mellitus (T2DB), have gained interest among both clinicians and medical researchers. Metformin belongs to the group of biguanide drugs that are mainly prescribed for insulin resistance (IR), for obesity, and for patients suffering from polycystic ovarian syndrome (POS) [1]. The general systemic effects of metformin involve the reduction of glucose production in the liver and increased insulin sensitivity in peripheral tissues. Metformin has also been proposed as a calorie restriction mimetic (CRM) that has been shown in combination with calorie restriction, to reduce morbidity and mortality in both healthy and tumorigenic mice, thus increasing overall lifespan [2].

In the field of regenerative medicine, major scientific interest is focused on the cytophysiological characteristics and clinical application of mesenchymal stem cells (MSCs). Those cells are found in many types of adult tissues, including bone marrow and adipose tissue [3]. Although adipose tissue-derived stem cells (ASCs) share similar characteristics with bone marrow mesenchymal stem cells (BMSCs), they have several advantages over BMSCs. They are easily accessible with minimal invasive harvesting procedure and the isolation yields a great number of cells, crucial for effective stem cell therapies and bone engineering.

Furthermore, it has been shown that metformin may inhibit the growth of tumor cells and thus may potentially find wide application in anticancer therapies [4–8]. Hirsch et al. [9] demonstrated that metformin inhibits cellular transformation and selectively kills cancer stem cells in genetically distinct breast cancer cells lines. The inhibitory effect of metformin on tumor cells* in vitro* is associated not only with its cytostatic properties, but also with proapoptotic actions in the tumor cells [10]. To date, the signaling pathways of metformin's mechanism of action have not been fully understood and require further study. However, it seems that metformin affects insulin-like growth factor (IGF) pathways by activation of 5′-adenosine monophosphate-activated protein kinase (AMPK), which plays a key role in insulin signaling and energy sensing [11]. Moreover, it has been shown that, in the context of atherosclerosis, metformin inhibits NF-*κ*B activation, decreases C-reactive protein levels [12], and inhibits the inflammatory response via a pathway involving AMPK and the tumor suppressor PTEN [13].

Moreover, recent studies have indicated antioxidative properties of metformin. Increased production of the intracellular level of reactive oxygen species (ROS) results from the imbalance of the antioxidant scavenger enzymes that metabolize free radicals. The antioxidant enzymes include manganese superoxide dismutase (MnSOD), copper and zinc (Cu/Zn) SOD, catalase, and glutathione peroxidase. When the level of ROS exceeds the defense mechanisms, a cell is said to be in a state of “oxidative stress” [14]. The enhanced production of ROS during environmental stresses can pose a threat to cells by causing peroxidation of lipids, oxidation of proteins, and damage to nucleic acids, ultimately leading to death of the cells. Thus dealing with excessive ROS may improve cellular metabolism and in consequence increase the lifespan. It was also shown that metformin exerted intracellular antioxidant properties by decreasing ROS production through the inhibition of protein kinase C activity [15]. Recent studies indicate that its primary effect is in mitochondria, where it interferes with respiratory complex I and reduces ATP production, leading to the activation of AMP kinase [16].

However, the potential role of metformin in reducing ROS in ASC is still not well established.

It has also been reported that metformin can induce MC3T3-E1 osteoblastic cell differentiation and bone matrix synthesis via AMPK activation and subsequent induction of endothelial nitric oxide synthase (eNOS) and bone morphogenetic protein-2 (BMP-2) expression [17, 18]. Metformin was also found to enhance rat osteoblasts proliferation, increase alkaline phosphatase activity, and increase the number of formed mineralized nodules. Our group recently showed that metformin, in a dose-dependent manner, affects the viability, morphology, and ultrastructure of mouse bone marrow-derived multipotent mesenchymal stromal cells and the Balb/3T3 embryonic fibroblast cell line [19]. We have also demonstrated that the expression of tandem gene H19/IGF2, as well as Oct4, a pluripotent stem cell marker, in part correlates with particular dosage application of metformin* in vitro*. Additionally, we found a significant correlation between the activity of the ASCs and osteopontin at the mRNA and protein level* in vitro* and* in vivo*. The mounting evidence indicates that the range of metformin actions may be significantly wider than once thought, and thus the application of metformin may open new avenues in the treatment of various medical conditions [20, 21].

Bone development is a complex and dynamic process involving several different mechanisms that maintain the balance between bone formation and bone resorption. This balance might be affected by different factors including physical activity, endogenous hormones, or drugs [22]. Essential to this balance and to the formation of the skeletal system is proosteoblastic differentiation of bone marrow stem cells, osteoclast activity, and the osteoblast-adipocyte relationship. Recently, it has been demonstrated that the complement system plays an important role in both bone development and homeostasis and specifically influences osteoblast and osteoclast activity [23]. Moreover, it has been shown that complement plays a role in the bone repair process, thus making a bridge between skeletal and immunological systems.

Bearing in mind that previous research has indicated a proosteogenic effect of metformin on BMSCs or commitment cell lines* in vitro*, we became interested in the effect of metformin on the osteogenic potential of mice, as well as the effects of metformin on bone* in vivo.* It has been shown that metformin prevents bone loss induced by ovariectomy in rats [24], suggesting that metformin confers a protective effect against bone loss. Further research on rats showed that 2 weeks of metformin supplementation led to increased trabecular volume, osteocyte density, and osteoblast number in femoral metaphysis [25]. Wang et al. [26], using insulin-resistant mice, also showed that 6 weeks of metformin administration protects femoral bone architecture [26]. However, little is known about the effects of metformin on bone trabecular architecture and bone density in healthy mice.

The aim of our study was to investigate the effects of metformin on ASCs osteogenic differentiation potential* in vitro* and on bone density and mineralization in healthy mice.

## 2. Materials and Methods

All reagents used in this experiment were purchased from Sigma-Aldrich (Poland), unless indicated otherwise.

### 2.1. Experimental Animals

The experiment was performed on 4-week-old C57BL/6 mice (*n* = 18). All mice were housed three per cage in an ultraclean facility on ventilated racks and were provided with food and water ad libitum during th experiment period. Mice received a standard diet with 4.2% fat (Morawski, Labofeed H, Poland) and were maintained on a 12-h light-dark cycle at 22 ± 0.2°C. The animals were purchased from the Animal Laboratory House, Wroclaw Medical School, and housed in the Animal Experimental Laboratory (Wroclaw Medical School, Wroclaw, Poland).

The animals used in the study were divided into two groups: (a) control mice receiving drinking water only [*n* = 9] and (b) the group receiving metformin in drinking water at concentration equal to 2.8 mg/day (Metformax® 850; Teva Pharmaceuticals, Poland) for 8 weeks [*n* = 9]. Water was changed every two days. After the experiment, the mice were fasted for 24 h. Body weight measurement was carried out using electronic scale (RADWAG PS/C1 series, Poland). Blood was collected from the animals using a cardiac puncture method. Biochemical analysis of blood samples, such as glucose and lipids measurements, was performed with Erba XL 300 platform (Erba Diagnostics Mannheim GmbH, Germany). After euthanasia of the animals, abdominal subcutaneous adipose tissue, as well as both tibia bones, was collected from each animal.

Cells or tissues obtained from animals treated with drug for 8 weeks are designated as the 8-week metformin (Met 8) group and from the control group were designated as control.

### 2.2. Isolation of Mice Adipose-Derived Mesenchymal Stem Cells (MuASCs) from Adipose Tissue

The MuASCs were isolated from subcutaneous adipose tissue of C57BL/6 mice (*n* = 9 Met 8 mice, *n* = 9 control mice). Cells were isolated using a protocol previously described by Grzesiak et al. [27]. Tissue samples were placed in sterile Hanks' balanced salt solution (HBSS), containing 1% of antibiotic/solution (P/S/A: Penicillin/Streptomycin/Amphotericin B) and washed extensively to remove contaminating debris and erythrocytes. After removing blood vessels, the tissue was cut into small pieces using surgical scissors. Aspirates were then transferred into sterile tubes and treated with collagenase type I enzyme (1 mg/mL) in PBS for 40 min at 37°C with gentle agitation. The obtained tissue homogenate was then centrifuged for 10 min at 1200 ×g. The supernatant was then discarded, while the cell pellet was resuspended in culture media and plated onto a conventional tissue culture flask.

### 2.3. Cells Culture

All stages of the experiment were performed under aseptic, standardized culture conditions (37°C, 5% CO_2_). Primary cultures were plated on T25 culture flasks in culture media consisting of Dulbecco's Modified Eagle's Medium (DMEM) with nutrient F-12 Ham, 10% of Fetal Bovine Serum (FBS), and 1% solution of P/S/A. The medium was changed every two days, while passage of cells was performed after reaching 80–90% confluence. Prior to the experiment, cells were passed three times using trypsin solution (TrypLE*™*; Life Technologies, Carlsbad, USA) in accordance with manufacturer's protocol.

### 2.4. Immunophenotyping of MuASCs and Multipotency Assay

Isolated cells were characterized by the expression of following markers: CD29, CD44, CD45, CD73, and CD105. Prior to staining, cells were cultivated onto 24-well dishes designated for immunofluorescence preparations. For each staining, cultures were run in triplicate. The following antibodies were used in the experiment: rabbit anti-mouse integrin-b-1 (CD29), dilution 1 : 100; rabbit anti-mouse CD44 (R&D Systems, Minneapolis, USA), dilution 1 : 100; rabbit anti-mouse CD45, dilution 1 : 100; rabbit anti-mouse NT5E (CD73), dilution 1 : 200; rabbit anti-mouse endoglin (CD105), dilution 1 : 200. Secondary antibody conjugated with atto-488 fluorescence label goat anti-mouse IgG atto-488 was diluted 1 : 400. Immunocytochemistry was performed in accordance with the manufacturer's instructions. Briefly, cells were incubated with certain primary antibodies overnight, while incubation with secondary antibodies lasted 1 hour and was performed in 37°C, in the dark. To assess the binding specificity of secondary antibodies, negative controls were included. To visualize cells nuclei, specimens were counterstained with 4′,6-diamidino-2-phenylindole (DAPI). The samples were imaged using an inverted fluorescence microscope (AxioObserverA1, Carl Zeiss, Jena, Germany) and captured with Canon PowerShot. Multipotency of MuASCs was confirmed by adipogenic and osteogenic differentiation of cells, cultured in commercially available media (StemPro®, Life Technologies) onto 24-well plates. Adipogenic differentiation was confirmed with Oil Red O, to visualize lipid droplets. Osteogenic differentiation was confirmed with Alizarin Red staining of the extracellular matrix mineralization. Experiments were carried out simultaneously and performed in triplicate.

### 2.5. Cell Viability and Colony Forming Efficiency Assay

For the assays, cells were plated onto 24-well plates and inoculated with a 0.5 mL volume of culture medium at a concentration of 2 × 10^4^ cells per well. The number of viable cells in the culture was evaluated using resazurin-based assay kit (Alamar Blue) in accordance with the manufacturer's instruction after 1, 2, 5, and 7 days of cell culture. Briefly, to perform the assay, the culture medium was collected and replaced with a medium containing 10% of the dye. Cells were then incubated for 2 hours at 37°C, followed by supernatant collection. Absorbance levels of the supernatants were measured spectrophotometrically using 96-well microplate reader (Spectrostar Nano, BMG Labtech, Ortenberg, Germany). The dye reduction was evaluated at a wavelength of 600 nm and a reference wavelength of 690 nm. Each test included a blank that consisted of complete medium with dye. The cell number was obtained from the test data and allowed to calculate the population doubling time (PDT), as well as proliferation factor (PF).

The calculated values of PF reflect the activity of the experimental group (Met 8) in relation to the control group, giving an arbitrary value equal to 1. A PF value higher than 1 indicated increased cellular activity, while a PF lower than 1 showed decreased proliferation potential. PDT was calculated using online software (http://www.doubling-time.com/).

To evaluate the ability of cells to form colonies, a colony forming efficiency (CFE) assay was performed. Cells were seeded onto 6-well plates at an initial density of 1 × 10^2^ per well and inoculated in culture media (DMEM with nutrient F-12 Ham, 10% FBS, 1% P/S/A). After seven days of propagation, cells were washed with 4% ice-cold paraformaldehyde and stained with pararosaniline. Colonies of more than 50 cells were counted, and the efficiency of colony forming was calculated using the formula presented as follows [28]:

(1) $$\begin{array}{c} \text{CFUfs}\left[\;\%\;\right]=\frac{\text{the number of colonies}}{\text{initial cell number}}\times 100\%. \end{array}$$

### 2.6. Evaluation of Cell Morphology, Ultrastructure, and Senescence Markers

Cell morphology, cellular composition, and culture growth pattern were analyzed using an inverted, fluorescence microscope (Axio ObserverA1, Zeiss, Jena, Germany) and a scanning electron microscope (SEM; EVO LS15, Zeiss, Jena, Germany).

The morphology of cells was evaluated on the 7th (standard culture), 12th (adipogenic stimulation), and 18th (osteogenic stimulation) days. Prior to observation, cells were cultured on 24-well plates. After fixation in 4% ice-cold paraformaldehyde (40 min), cells membranes were permeabilized using 0.1% Triton X-100 for 15 min. After washing with HBSS, cells were stained with atto-565 labeled phalloidin for 40 min to visualize actin filaments. Cells were then washed again with HBSS three times and the nuclei were subsequently counterstained with diamidino-2-phenylindole (DAPI) for 5 min. During fluorescence staining, cells were incubated at room temperature in the dark.

To confirm osteogenesis and adipogenesis, Alizarin Red and Oil Red O staining were applied, respectively. First the culture media were removed, and the cells were then washed with HBSS followed by treatment with 4% ice-cold paraformaldehyde for 10 min. To visualize the extracellular mineralized matrix, cells were incubated with 2% Alizarin Red solution for 10 min. After dye discarding, cells were washed a few times with distilled water. For intracellular lipid droplets visualization, paraformaldehyde was replaced with 60% isopropanol and the cells were incubated with it for 5 min. Then the cells were rinsed with distilled water and Oil Red O solution was added for 15 min, followed by hematoxylin counterstaining for 1 min. Images were captured using a Canon PowerShot camera.

Detailed cells morphology was evaluated using SEM. Cells were rinsed with distilled water and dehydrated in a graded ethanol series (concentrations from 50% to 100%, every 5 min). Samples were then sprinkled with gold (ScanCoat 6, Oxford), transferred to the microscope chamber, and observed using SE1 detector, at 10 kV of filament tension. Additionally, the number of bone nodules, the size of the nodules, and the calcium and phosphorus concentration within the nodules were analyzed by SEM with energy dispersive X-ray analysis (SEM/EDX). A QUANTAX detector (Brüker, Billerica, USA) with 10 kV of filament tension was used to perform a line scan analysis of randomly selected cells. The obtained values of calcium and phosphorus deposition were presented as weight percentage (wt%).

To evaluate amounts of viable and dead cells, a Cellstain Double Staining Kit was applied. Live cells were stained with Calcein-AM and emitted a green fluorescence signal, whereas dead cell nuclei were stained with Propidium Iodide and emitted a red/orange fluorescence. Cells were then observed using fluorescence microscopy. All procedures were performed in accordance with the manufacturer's instructions. Immunofluorescence staining for caspase-3 was performed using anti-caspase 3 antibodies (anti-caspase-3 active antibody produced in rabbit, cat number C8487). Prior to investigation of senescence-associated *β*-galactosidase (*β*-gal), cells were stained using a commercially available kit (Senescence Cells Histochemical Staining Kit) following manufacturer's protocol.

### 2.7. Analysis of Oxidative Stress and Superoxide Dismutase Activity (SOD)

To evaluate stress levels, the cells were cultured in medium without phenol red for 7 days and the tests were performed on the 7th day. Nitric oxide (NO) concentration was assessed with the Griess Reagent Kit (Life Technologies), superoxide dismutase (SOD) was assessed by SOD Assay Kit, and the level of reactive oxygen species (ROS) was estimated with a 5-(and-6)-chloromethyl-2′,7′-dichlorodihydrofluorescein diacetate (H2DCF-DA, Life Technologies) solution. All procedures were performed in duplicate in accordance with the manufacturer's protocols.

### 2.8. Evaluation of Osteogenic and Adipogenic Differentiation Potential of MuASCs

For the* in vitro* osteogenic and adipogenic assays, cells were planted onto 24-well plates at an approximate density of 2 × 10^4^ cells per well. Prior to osteogenic differentiation of MuASCs, cells were cultured in STEMPRO® Osteogenesis Differentiation Kit (Life Technologies, Carlsbad, USA) for a period of 18 days. For adipogenic differentiation, cells were cultured in STEMPRO® Adipogenesis Differentiation Kit (Life Technologies, Carlsbad, USA) for 12 days. The differentiation media were supplemented with 1% of P/S/A and changed every two days. Cultures expanded in standard culture media (DMEM with nutrient F-12 Ham, 10% FBS, 1% P/S/A) served as a control (nonstimulated), which allowed determining effectiveness of the differentiation process. All procedures were performed in accordance with the manufacturer's instructions.

### 2.9. Detection of Extracellular Form of Osteopontin (OPN), Osteocalcin (OCL), and Bone Morphogenetic Protein-2 (BMP-2) with Enzyme-Linked Immunosorbent Assay (ELISA)

The investigated markers were detected in the supernatants collected at the last day of the differentiation process, the 18th day of osteogenic stimulation and the 12th day of adipogenic stimulation. The presence of osteopontin (OPN) was determined using Mouse/Rat Osteopontin Quantikine ELISA Kit (R&D Systems, Minneapolis, USA), whereas osteocalcin (OCN) was assessed with the Mouse Gla-Osteocalcin High Sensitive EIA Kit (Takara, Otsu, Japan). The level of bone morphogenetic protein (BMP) was investigated with the Mouse BMP-2 Quantikine ELISA Kit (R&D Systems, Minneapolis, USA). All procedures were performed according to the manufacturer's protocol. The amount of detected proteins was expressed as ng/mL of supernatant.

### 2.10. Analysis of Gene Expression: Real-Time Reverse Transcription Polymerase Chain Reaction (qRT-PCR) of OPN, BMP-2, and Adiponectin (ADPQ)

After osteogenic stimulation, on the 18th day of culture, the cultures were rinsed with HBSS and lysed in a culture dish with 0.5 mL of TRI Reagent®. The total RNA was isolated using a phenol chloroform method. The resulting samples were then diluted in DEPC-treated water. The quantity and quality of the total RNA were determined using a nanospectrometer (VPA biowave II). To remove any traces of genomic DNA, samples were digested using DNase I RNase-free kit (Thermo Scientific, Waltham, USA). Each reaction was performed with 100 ng of total RNA, which was then transcribed to complementary DNA (cDNA) with the Moloney murine leukemia virus reverse transcriptase (M-MLV RT), provided with the Tetro Reverse Transcriptase kit (Bioline, London, UK). Both RNA purification and cDNA synthesis were performed following the manufacturers protocol, using a T100 Thermo Cycler (Bio-Rad, Hercules, USA). Quantitative RT-PCR was performed in a total volume of 20 *μ*L using the SensiFast SYBR & Fluorescein Kit (Bioline, London, UK). The primer concentration in each reaction equaled 500 nM, and the primer sequences used for each specific reaction are listed in Table 1. The analysis was performed using CFX ConnectTM Real-Time PCR Detection System (Bio-Rad, Hercules, USA). To confirm the specificity of the reaction products, an analysis of the dissociation curve was performed. The value of the threshold cycle (Ct) was used to calculate the fold change in relation to the expression of the housekeeping gene, B2M (beta-2 microglobulin).

### 2.11. *Ex Vivo* Analysis of Bone Mineral Concentration and Density by Microtomography (*μ*CT) and SEM-EDX Calcium Deposition Analysis

Prior to performing *μ*CT analysis, the bone samples were fixed in 4% formaldehyde in PBS for 48 h. Specimens were then dehydrated in an increasing ethanol gradient of 50%, 60%, 70%, 80%, 96%, and 100% and then analyzed in *μ*CT.

The tomographic studies were carried out in the Laboratory of Micro and Nano Tomography (LMINT), Faculty of Physics and Applied Computer Science, AGH University of Science and Technology, Krakow, Poland. The *μ*CT measurements were performed using a Nanotom 180N (GE Sensing & Inspection Technologies, Phoenix X-ray GmbH). The machine is equipped with a nanofocus X-ray tube with a maximum 180 kV voltage. The tomograms were registered using a Hamamatsu 2300 × 2300 pixel 2D detector. The reconstruction of measured objects was performed using datosX ver. 2.1.0 (GE) with the use of a Feldkamp algorithm for cone beam X-ray CT [29]. The postreconstruction data treatment was performed using VGStudio Max 2.1 [30] and free Fiji software [31] with the BoneJ plugin [32].

All examined specimens were scanned at 70 kV of source voltage and 200 *μ*A, with a 360-degree rotation of the specimen in 1400 steps. The exposure time was 500 ms, with a frame averaging of 3 and image skip of 1 applied, resulting in a scanning time of 60 minutes. The reconstructed images had a voxel size of 5 *μ*m^3^.

For SEM-EDX analysis, the femoral bones were fixed in 2.5% glutaraldehyde for one hour, washed in distilled water, dehydrated in a graded ethanol series, sputtered with carbon (ScanCoat 6, Oxford), and imaged using an SEM at 10 kV of filament's tension. Additionally, specimens were prepared for EDX analysis according to the method described previously by our laboratory [33]. Observations were performed at a filament tension of 10 kV. The detection of mineralized matrix was performed using SEM combined with EDX (Bruker, Coventry, UK) to analyze the surface distribution of calcium and phosphorus. The values obtained were presented as weight percentage (wt%).

### 2.12. Statistical Analysis

All experiments were performed with *n* = 3 or more. Statistical analysis was performed using GraphPad Prism 5 software (La Jolla, USA). Differences between groups was determined using one-way analysis of variance (ANOVA) with* post hoc* Tukey's multiple comparison or Student's *t*-test. Differences with a probability of *p* < 0.05 were considered significant.

## 3. Results

The clinical characteristics of the C57BL/6 mice included in the study, assessed following the 24 h of fasting at the end of the experiment, are presented in Table 2.

### 3.1. Immunophenotyping Characterization of Isolated MuASCs and Multipotency Assay

**Figure 1:**
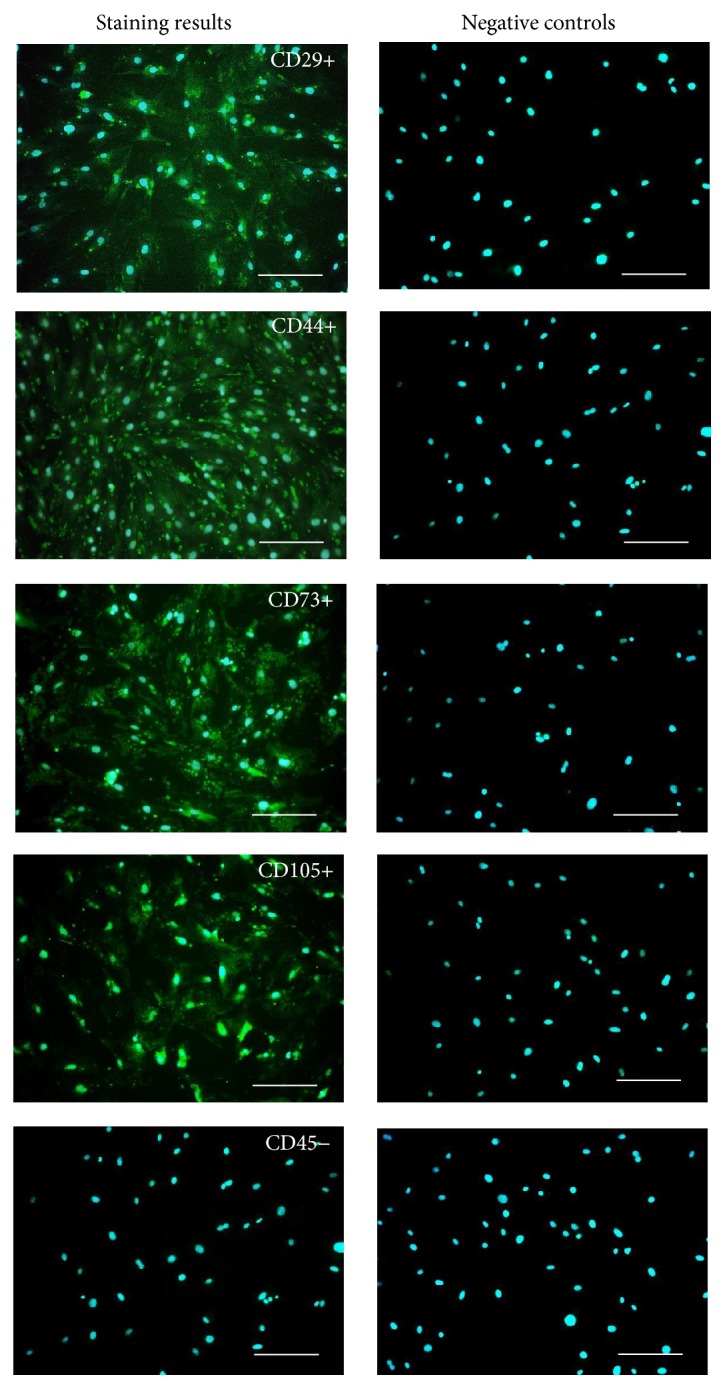
Results of MuASCs immunophenotyping. Obtained cells were positive for the mesenchymal markers CD29, CD44, CD73, and CD105 and show lack of expression for the hematopoietic marker CD45. Each marker was stained with a specific antibody and secondary antibodies conjugated with atto-488; nuclei were counterstained with DAPI. Magnification ×100, scale bar: 250 *μ*m.

**Figure 2:**
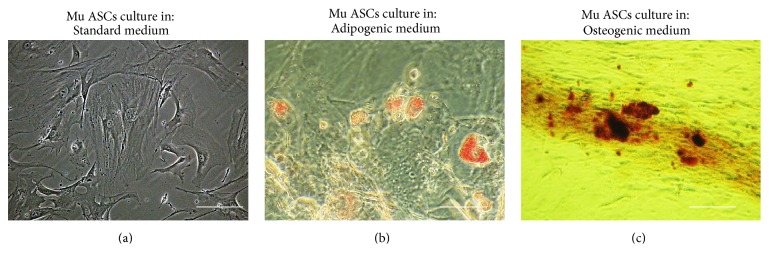
Results of multipotency assay. Morphology of MuASCs cultured in standard (a), adipogenic (b), and osteogenic (c) conditions. Calcium deposits in mineralized extracellular matrix were visualized with Alizarin Red staining, while lipid droplets formed in adipogenic stimulation with Oil Red O. The images included in the figure were chosen to be representative. Magnification ×100, scale bar: 250 *μ*m.

In order to confirm multipotent character of isolated ASCs, immunohistochemical staining and multipotency assays were performed. Immunohistochemical staining revealed that MuASCs population did not express CD45 hematopoietic marker (Figure 1). In contrast, we observed positive reactions for mesenchymal markers which are specific to multipotent stromal cells including CD29, CD44, CD73, and CD105 (Figure 2). Moreover, the multipotent character of isolated MuASCs was confirmed by positive adipogenic and osteogenic differentiation (Figure 2). Formation of extracellular matrix mineralization was visualized by Alizarin Red staining (Figure 2(c)). In addition, Oil Red O staining was performed to reveal the formation of intracellular lipid droplets (Figure 2(b)). Cells cultured in standard medium served as a control (Figure 2(a)).

### 3.2. Proliferation Factor (PF), Population Doubling Time (PDT), and Colony Forming Efficiency (CFE) of MuASCs during Seven-Day Viability Test

**Figure 3:**
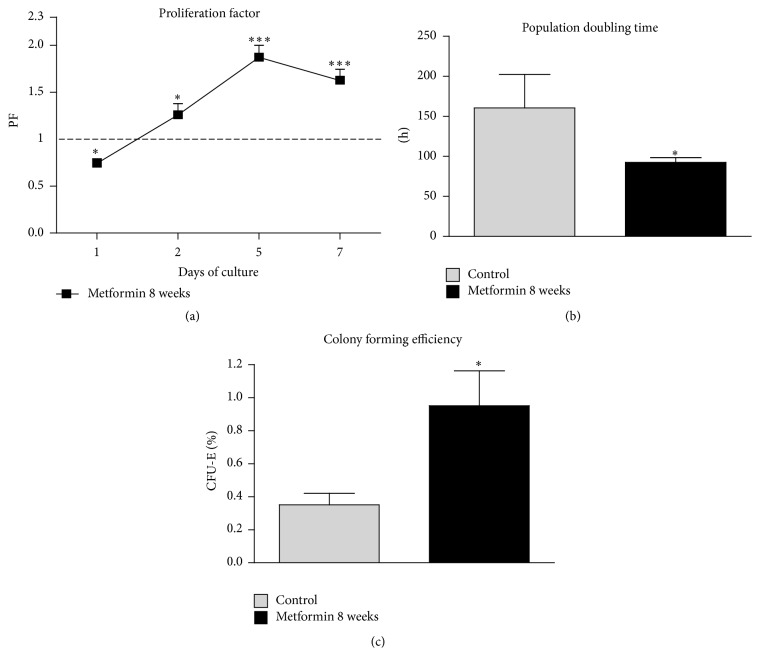
Seven-day viability test of MuASCs. Growth kinetics and proliferation potential of MuASCs during seven-day culture. Proliferation factor calculated in comparison to control group (a). Time required for doubling the cell population expressed in hours (b), and the efficiency to form single cell derived colonies (c). Results expressed as mean ± SD. ^*∗*^ *p* value < 0.05, ^*∗∗∗*^ *p* value < 0.001.

The proliferation of MuASCs was evaluated after 1st, 2nd, 5th, and 7th days of culture. The viability characteristics of MuASCs including PF, PDT, and CFE were assessed in comparison to the control group. The analysis of cytophysiological activity of the Met 8 cells showed proliferation activity that was decreased after 24 hours of propagation. However, this was followed by a significantly accelerated proliferation on the 2nd, 5th, and 7th day of culture. The highest PF was recorded on 5th day (*p* < 0.001) of experimental culture (Figure 3(a)).

The proliferation assay analysis showed that metformin taking contributed to the change in PDT. The control group reached PDT in 160.4 ± 41.9 h. Interestingly, the time needed to double the population of MuASCs was considerably reduced in Met 8 group (*p* < 0.05), which was only 92.58 ± 5.8 h (Figure 3(b)).

ASCs plated on low density tend to form colonies originated from a single cell. It was found that cells from the Met 8 group displayed a significantly (*p* < 0.05) higher CFE than the control group (Figure 3(c)).

### 3.3. Evaluation of MuASCs Morphology

**Figure 4:**
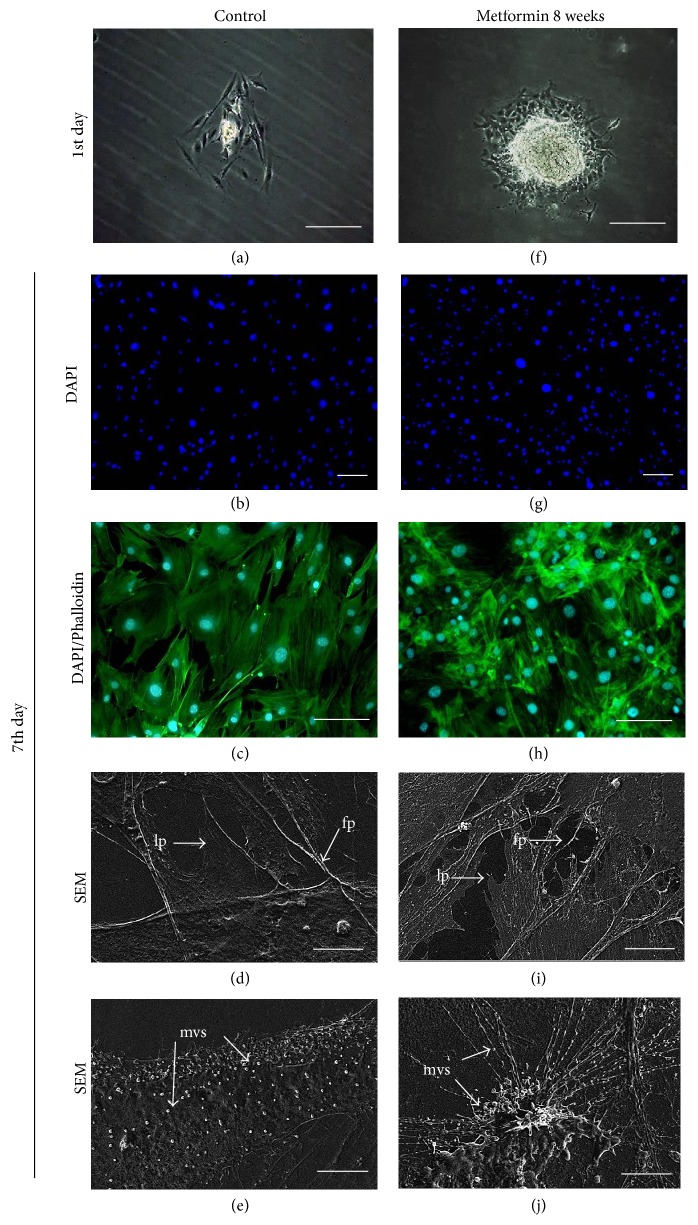
Morphology and cellular composition of MuASCs evaluated on 7th day of culture; light microscope (a, f), staining for DAPI (b, g), DAPI/phalloidin merged (c, h), and SEM (d, e, i, j) images. Morphological features were indicated with proper abbreviations: fp: filopodia, lp: lamellipodia, and mvs: microvesicles. Magnification: DAPI ×50, scale bar: 250 *μ*m; DAPI/phalloidin ×100, scale bar: 250 *μ*m; SEM ×5000, scale bar 2 *μ*m.

During the initial stage of culture (from day one) we observed the formation of embryonic-like nodules (Figures 4(a) and 4(f)), which displayed the largest morphology in the Met 8 group. The control culture of MuASCs was characterized by large, flat, and irregularly shaped cells. The cytoskeleton of those cells was well developed, and DAPI staining revealed centrally positioned nuclei (Figures 4(b) and 4(c)). SEM pictures revealed few actin projections, filopodia and microvesicles (MVs) that were located near to nucleus, as well as on the edge of the cell body (Figures 4(d) and 4(e)).

In the Met 8 group, we observed more cells with a fibroblast-like shape and the embryonic-like nodules were of greatest size. Moreover, the size of the cells seemed to be smaller. No apoptotic bodies were observed and the actin cytoskeleton network was well organized (Figures 4(g) and 4(h)). The number of cells was visibly higher than in the other groups. The SEM imaging allowed visualization of multiple, thin cellular projections (Figure 4(i)) and rich production of MVs (Figure 4(j)).

### 3.4. Evaluation of Senescence Markers

**Figure 5:**
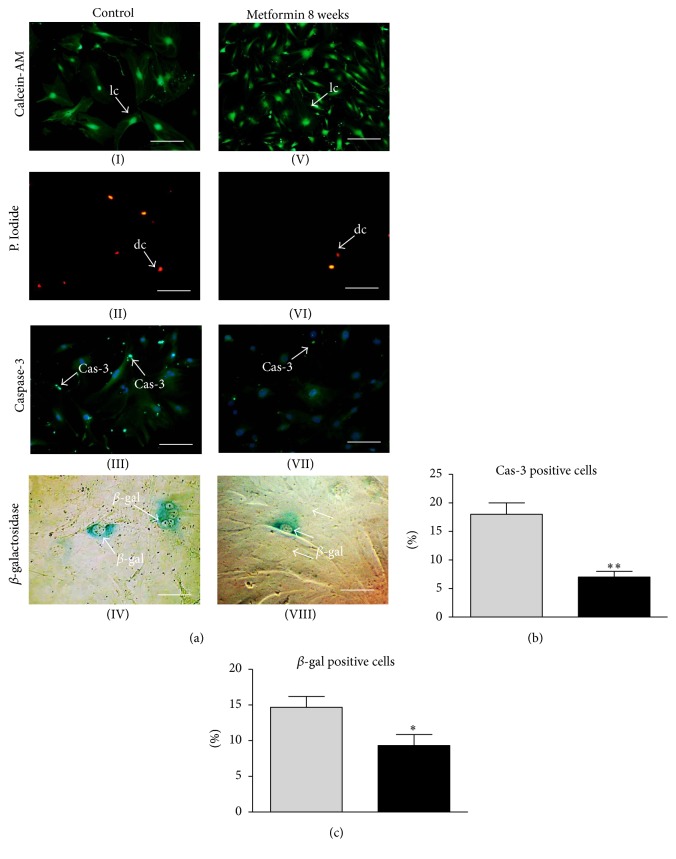
Evaluation of senescence markers in MuASCs after 7th day of culture. Photographs showing different types of cellular senescence-associated markers. Cells stained with Calcein-AM (I, V), Propidium Iodide (II, VI), antibodies for caspase-3 (III, VII, nuclei counterstained with DAPI), and senescence dye (IV, VIII) showing cells with *β*-galactosidase accumulation. Abbreviations used to describe different cell types: lc: live cell, dc: dead cell, cas-3: caspase-3 positive cell, and *β*-gal: *β*-galactosidase positive cell. Magnifications: Calcein-AM and P. Iodide ×50, scale bar: 500 *μ*m; caspase-3 ×100, scale bar: 250 *μ*m; *β*-galactosidase ×200, scale bar: 125 *μ*m. Data was also presented quantitatively by calculating percentage of cells positive for caspase-3 (b) and *β*-gal (c). Results expressed as mean ± SD. ^*∗*^ *p* value < 0.05, ^*∗∗*^ *p* value < 0.01.

To evaluate the percentage of dead cells in each group, we used a Cellstain Double Kit. Calcein-AM/Propidium Iodide staining revealed that the number of dead cells was significantly reduced in the Met 8 group (Figure 5(a)(V, VI)) in comparison to the control (Figure 5(a)(I, II)). To determine if a decrease in cell growth was connected to an increased apoptosis level, we examined the activity of caspase-3 by immunofluorescence technique. The obtained pictures showed that the greatest fluorescence signal was in the control group (Figure 5(a)(III)), with the signal decreasing in the Met 8 group (Figure 5(a)(VII)), where the lowest activity of caspase-3 was observed.

We also performed staining for senescence-associated *β*-galactosidase (*β*-gal). The amount of blue, *β*-gal positive cells was higher in the control (Figure 5(a)(IV)) than in the Met 8 group, where we noticed a reduction in the number of *β*-gal positive cells (Figure 5(a)(VIII)).

Moreover, to provide quantitative data, percentage of cells positive for both caspase-3 (Figure 5(b)) and *β*-gal (Figure 5(c)) was calculated. Both markers were decreased in Met 8 group.

### 3.5. Oxidative Stress Accumulation and SOD Activity

**Figure 6:**
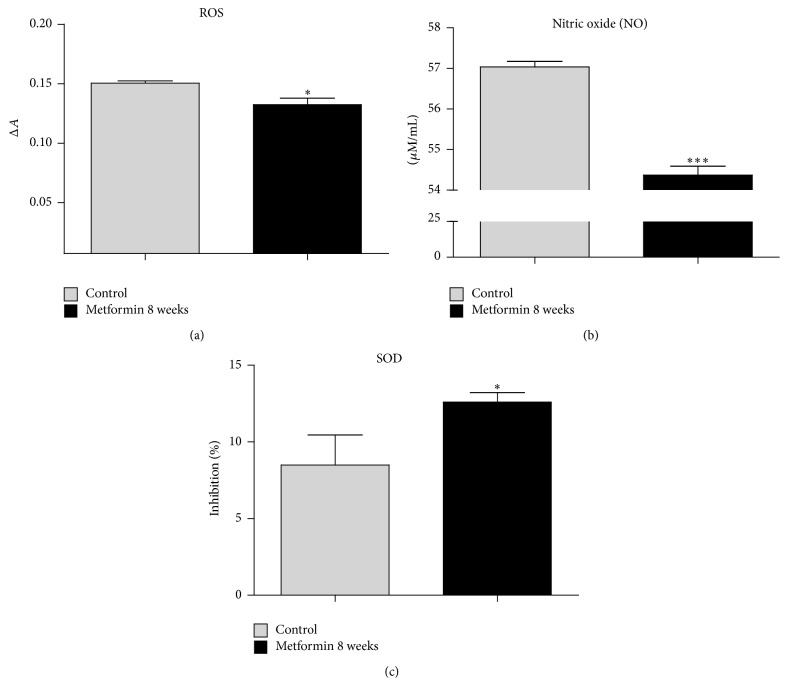
Assessment of oxidative stress and SOD activity in MuASC. To investigate whether treatment with metformin affects the oxidative status of MuASC, we investigated the extracellular levels of ROS (a) and NO (b). Moreover, the activity of antioxidant enzyme SOD was evaluated (c). Obtained data indicates that metformin supplementation decreases the oxidative stress and simultaneously improves the antioxidative protection coming from SOD. Results expressed as mean ± SD. ^*∗*^ *p* value < 0.05, ^*∗∗∗*^ *p* value < 0.001.

To evaluate the effect of metformin on accumulation of oxidative stress in MuASCs we investigated the levels of reactive oxygen species (ROS) and nitric oxide (NO) in those cells. The amount of both, ROS (Figure 6(a)) and NO (Figure 6(b)), was significantly decreased in cells obtained from animals treated with metformin (*p* < 0.05 and *p* < 0.001, resp.). To investigate the antioxidative defense of MuASC, the activity of superoxide dismutase (SOD) was assessed. We observed that metformin supplementation positively affects the activity of SOD, as it was significantly increased in Met 8 group (Figure 6(c), *p* < 0.05).

### 3.6. Proliferation Factor (PF) and PDT during Osteogenic Differentiation

**Figure 7:**
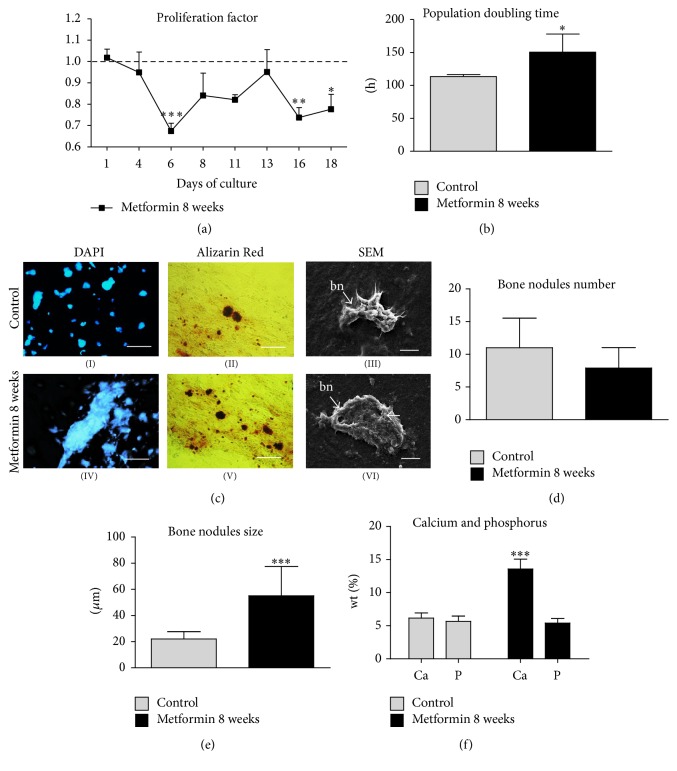
Evaluation of MuASCs osteogenic potential. Growth kinetics and morphology of cells cultured under osteogenic conditions. Proliferation factor (a), population doubling time (b). Morphology evaluation after 18 days of culture (c). DAPI staining (I, IV), Alizarin Red (II, V), and SEM pictures (III, VI). bn: bone nodule. Mean bone nodules number (d) and size (e). Evaluation of calcium and phosphorus depositions (f). Magnifications: DAPI and Alizarin Red ×100, scale bars: 250 *μ*m; SEM ×5000, scale bar: 5 *μ*m. Results expressed as mean ± SD. ^*∗*^ *p* value < 0.05, ^*∗∗*^ *p* value < 0.01, ^*∗∗∗*^ *p* value < 0.001.

The PF, as well as the PDT, was analyzed during the 18-day period of culturing MuASCs in osteogenesis-inducing medium. Obtained data showed that metformin supplementation influenced both the PF and PDT. The proliferation of MuASCs from the Met 8 group was decreasing during the first six days of propagation and reached the lowest value on the 6th day (*p* < 0.001). After that, the proliferation activity was stabilized. Significant differences from controls were observed only later, on the last two days of the experiment, the 16th (*p* < 0.01) and 18th (*p* < 0.05) days (Figure 7(a)).

The estimation of PDT showed that the time needed to double the population in the Met 8 group was significantly increased (*p* < 0.05) in comparison to control culture (Figure 7(b)).

### 3.7. The Morphology of Osteoblasts Originated from MuASCs

After osteogenic induction, cell morphology shifted from typical fibroblast-like cells to more flattened and cuboidal cells. Moreover, cytoskeletal projections became shorter. After 18 days of propagation, bone nodules became visible and showed strong black/red signal after Alizarin Red stain was applied (Figure 7(c)(II, V)). Characteristic osteoblast-like cells were observed in each of the experimental groups; however, the cells in Met 8 group displayed the most effective osteogenesis (Figure 7(c)(IV, V, VI)). In this group, bone nodules were the largest in size, and Alizarin Red dye absorption was most abundant.

The lowest efficiency of osteogenic induction was observed in control group, where nodule diameters were much smaller, and calcium deposits absorbed a smaller amount of Alizarin Red dye (Figure 7(c)(I, II, III)). Interestingly, in the Met 8 group we observed the lowest number of bone nodules (Figure 7(d)); however, they reached the greatest size (Figure 7(e)). Furthermore, calcium deposition was significantly higher in the Met 8 group, compared to the control group (Figure 7(f), *p* < 0.001). The obtained data suggest that metformin supplementation increases MuASCs osteogenic differentiation potential and increases its effectiveness.

### 3.8. Extracellular Identification of OPN, OCN, BMP-2, and RT-PCR for OPN and BMP-2

**Figure 8:**
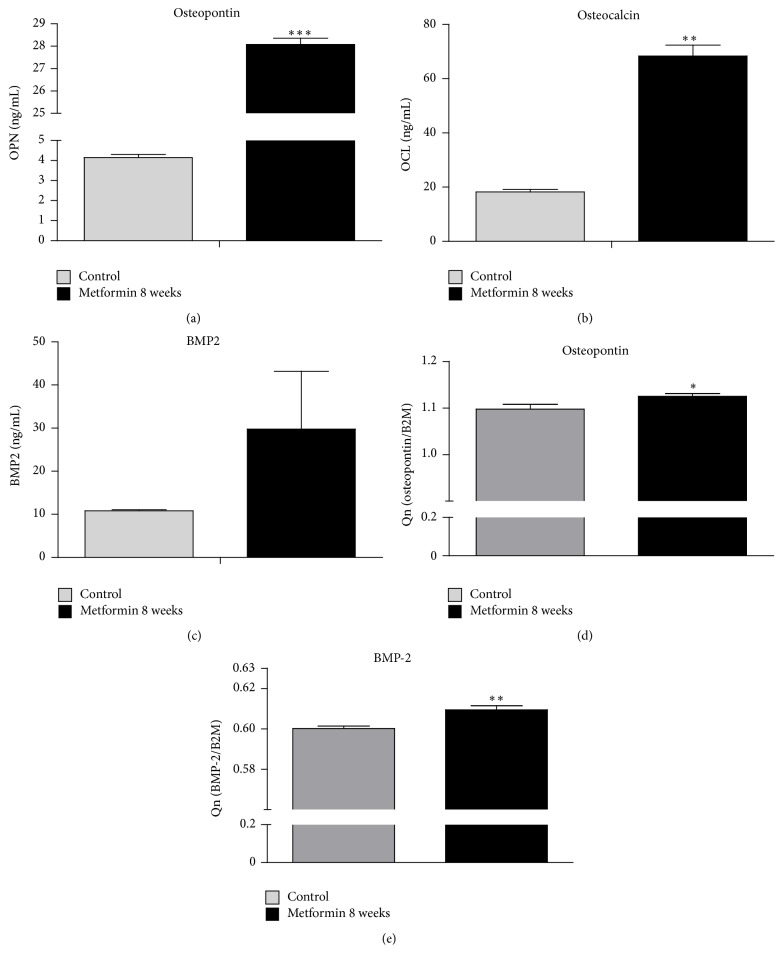
Concentration of osteogenic-related proteins and reverse transcriptase-polymerase chain reaction (RT-PCR) results. Extracellular protein levels under osteogenic conditions. Quantitative ELISA results for osteopontin (a), osteocalcin (b), and BMP-2 (c). Gene expression in MuASCs cultured in osteogenesis-promoting conditions. Osteopontin (d) and BMP-2 (e). Results expressed as mean ± SD. ^*∗*^ *p* value < 0.05, ^*∗∗*^ *p* value < 0.01, and ^*∗∗∗*^ *p* value < 0.001.

On the 18th day of osteogenic stimulation, quantitative analysis of extracellular OPN showed increased levels in the Met 8 (Figure 8(a), *p* < 0.001) group in comparison to the control culture. Like OPN, OCN concentration was also affected by the metformin supplementation. The OCN concentration was increased in the Met 8 (*p* < 0.01) group (Figure 8(b)). BMP-2 was also increased in Met 8 group, although without statistical significance (Figure 8(c)). Moreover RT-PCR analysis revealed increased mRNA levels of both OPN (Figure 8(d), *p* < 0.05) and BMP-2 (Figure 8(e), *p* < 0.01) in Met 8 group.

### 3.9. Proliferation Factor (PF), PDT, Morphological Changes, and ADPQ Expression during Adipogenic Differentiation

**Figure 9:**
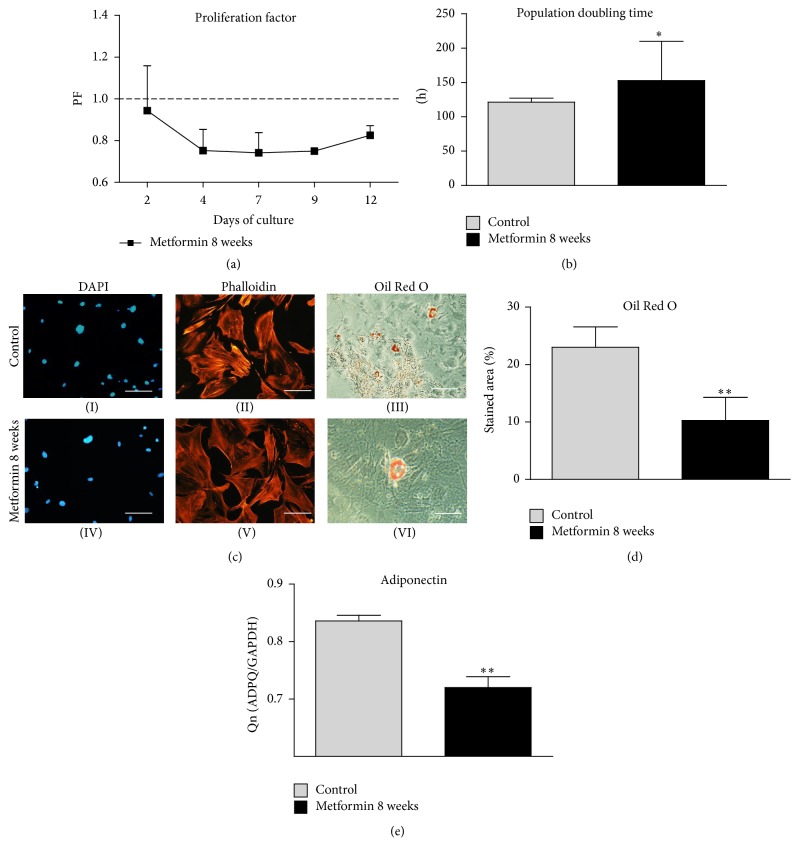
Evaluation of adipogenic potential. Growth kinetics and morphology of cells cultured under adipogenic culture conditions. Proliferation factor (a), population doubling time (b). Morphology evaluation after 12 days of culture (c). Nuclei staining with DAPI (I, IV), phalloidin (II, V), and Oil Red O (III, VI). Magnifications: DAPI/phalloidin, ×100, scale bars: 250 *μ*m; Oil Red O ×50, scale bar: 500 *μ*m. Oil Red O staining was quantified by calculation percentage of stained area (d). Moreover, using RT-PCR mRNA levels of Adiponectin were assessed (e). Results expressed as mean ± SD. ^*∗*^ *p* value < 0.05, ^*∗∗*^ *p* < 0.01.

The PF, as well as the PDT, was estimated during a 12-day period of culturing cells in adipogenic-inducing medium. MuASCs from Met 8 group showed decrease in PF compared to the control group (Figure 9(a)). The highest PF value was observed on the 2nd day, and from that time point a general decreasing tendency was observed. The PDT was also increased in metformin group and showed a significant difference (*p* < 0.05) in comparison to the control culture, as it was reduced by approximately 35% (Figure 9(b)).

Adipogenic differentiation was demonstrated by the accumulation of intracellular lipid droplets, indicated by Oil Red O staining on 12th day of culture (Figure 9(c)(III, VI)). In the control group, we observed the greatest amount of lipid droplets. The ratio of red staining area was significantly decreased in metformin group, where adipocytes displayed a smaller size and were reduced in number (Figure 9(c)(VI)). Furthermore, DAPI (Figure 9(c)(I, IV)) and phalloidin (Figure 9(c)(II, V)) staining revealed that cells from the control group became enlarged and spread out and simultaneously decreased the number of cytoskeletal projections. Moreover, we calculate percentage of area stained with Oil Red O (Figure 9(d)) and it was significantly reduced in Met 8 group (*p* < 0.01). Using RT-PCR we also assessed the expression of adiponectin mRNA, which was reduced in Met 8 group (Figure 9(e), *p* < 0.001). The obtained data suggests that metformin inhibits adipogenic differentiation of MuASCs.

### 3.10. *Ex Vivo* Analysis of Bone Mineral Concentration and Density

**Figure 10:**
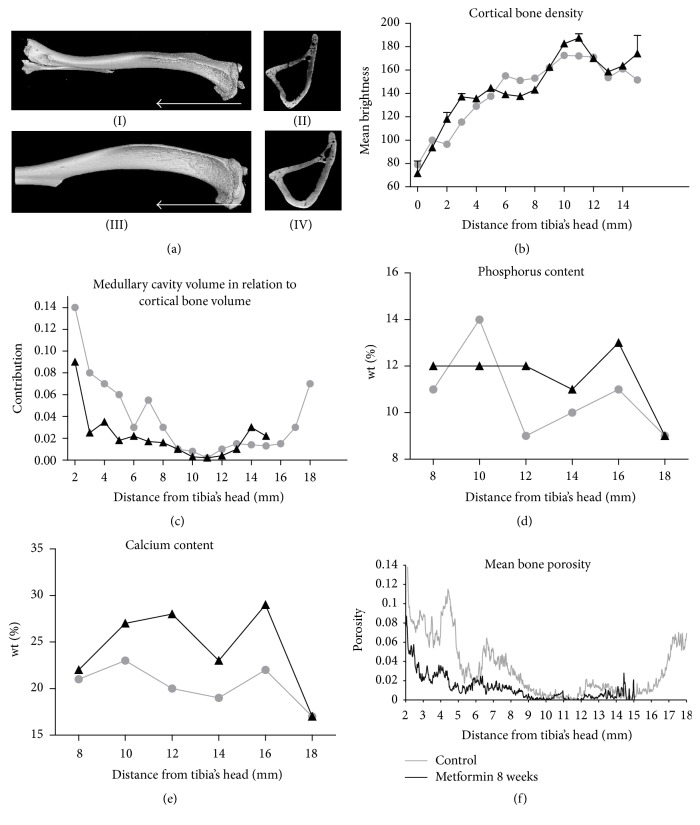
Nano-CT and SEM-EDX analysis. Nano-CT images of tibia bones and their transverse sections from control (I, II) and Met 8 (III, IV) mouse. White arrows indicate direction of performed scanning. Quantification of cortical bone density (b) and medullary cavity volume (c) in mice tibias by Nano-CT. SEM-EDX assessment of calcium (d) and phosphorus (e) content in tibia from control and Met 8 group. Mean bone porosity assessed by Nano-CT in the studied groups (f). Results expressed as mean, *n* = 5.

The morphology of murine tibia bones assessed by Nano-CT is shown in Figure 10(a): control group (I, II) and Met 8 (III, IV), respectively. Using Nano-CT, we also evaluated bone density on certain parts of the bone (scanning began from the proximal epiphysis, in the direction marked in Figure 9 by the white arrows). The greatest bone density was observed in Met 8, especially in the proximal 1–6 mm area of epiphysis, in comparison to other groups (Figure 10(b)). The smallest medullary cavity observed in the Met 8 group was at a distance between 2 and 7 mm, which indicates higher bone volume in this section (Figure 10(c)). Calcium concentration in the tibia was greater in Met 8 (Figure 10(d)) and, similarly, phosphorus deposition (Figure 10(e)). Moreover, we observed increased tibia porosity in the control group, especially at a distance between 4 and 5 mm (Figure 10(f)).

## 4. Discussion

Recently, more emphasis is being paid on investigating the multidirectional action of metformin* in vivo* and* in vitro*. While commonly known as an antidiabetic drug, metformin is recognized not only as a hypoglycemic agent, but also as a potential geroprotector, a drug that protects against the aging process [34]. It has been previously demonstrated that metformin increases the lifespan of mice [2] and enhances the viability and proliferative activity of mesenchymal stromal stem cells [35]. Thus, its application as an antiaging drug in part follows the hypothesis that “we are as old as our adult stem cells are.” The antiaging effects of metformin are most likely explained by the upregulation of circulating insulin-like growth factor-1 (IGF-1) in peripheral blood that has been recognized as a crucial factor in the context of longevity [36]. Bearing in mind the fact that mesenchymal stem cells are also partially linked with longevity by their ability to divide, proliferate, and eventually differentiate to replace a damaged tissue [37], it is reasonable to investigate the effects of metformin on the differentiation potential of endogenous MSCs, derived from metformin-treated animals.

The aim of the present study was to investigate how metformin administration affects the osteogenic and adipogenic differentiation potential of ASCs isolated from healthy mice that previously received oral metformin supplementation for 8 weeks, with a dose that is recommended for mice with DT2. Additionally, bearing in mind the divergent data concerning the effects of metformin on bone mineral density and bone regenerative ability, we also focused on the evaluation of bone density of healthy mice (without performing bone defects) treated with metformin. We found that ASCs derived from the 8-week metformin- (Met 8-) treated animals present different osteogenic and adipogenic differentiation potentials, with cells obtained from the Met 8 group presenting with more osteogenic than adipogenic character.

As observed in our study, metformin administration has an important influence on ASCs morphology, proliferation rate, and osteogenic/adipogenic differentiation ability, all of which are essential features of tissue regeneration. Recently published data by our group revealed that metformin affects stromal stem cells' proliferative activity in a dose-dependent manner [19]. We demonstrated that metformin increases the proliferative activity of BMSCs and mouse embryonic fibroblast cell line Balb/3T3 after 48 h of culture, when 1 mM of metformin supplementation was used [19]. Gao et al. [38] have demonstrated that BMSCs treated* in vitro* with 100 *μ*M metformin responded with an increase in proliferative activity. In turn, Śmieszek et al. [19] demonstrated that ASCs, compared to BMSCs, are more sensitive to metformin administration* in vitro* and thus even lower concentrations of 1 mM metformin resulted in decreased ASCs proliferative activity. Moreover, it was previously demonstrated that metformin administered orally through 5 weeks at a dose of 100 mg/day decreases expression of Ki-67 and osteopontin (OPN) in subcutaneous adipose tissue. The inhibitory effect of metformin on stromal stem cells has been explained by the activation of adenosine 5-monophosphate-activated protein kinase (AMPK) and inhibition of multiple molecular signaling pathways, which are important in protein synthesis control [33, 39].

In the current study, we found that prolonged administration of metformin (8 weeks) at a concentration equal to 2.8 mg/day in mice increases the viability and proliferative activity of ASCs and shortens their PDT. Simultaneously, we observed decreased activity of the senescence markers caspase-3 and *β*-galactosidase in ASCs isolated from the Met 8 group in comparison to the control group cells. Thus, metformin supplementation may be a potential option for overcoming the detrimental effects of aging on ASCs and might be considered in the pretransplantation period, especially in older patients, to enhance the effects of ASC based regenerative therapies.

Furthermore, we found that ASCs derived from Met 8 group were characterized by elevated synthesis of membrane-derived vesicles (MVs) observed during electron microscopy. As previously demonstrated by Ratajczak [40], MVs are rich in a broad range of growth factors that supports regenerative processes. MVs have also been demonstrated to be involved in intercellular signaling, and thus the elevated secretion of MVs by the cells derived from the Met 8 group might lead to improvement of regenerative abilities. Besides elevated MVs secretary activity, the ASCs derived from the Met 8 group were characterized by the development of extensive network of filopodia that transferred the MVs between the cells, as well as a robust net of lamellipodia. These structures play a crucial role in promoting both cell migration and extension. Moreover, filopodia are involved in cellular processes like wound healing and guidance towards a chemoattractant, which makes them a valuable characteristic of cells to be used for regenerative medicine [41]. Overall, our obtained data indicated elevated cytophysiological activity of ASCs derived from the Met 8 group that, from a regenerative point of view, seems to be fundamental.

Moreover, to investigate whether metformin administration affects the oxidative status of MuASC, we evaluate the levels of ROS and NO in those cells. Beyond its antidiabetic activity justifying its use in the treatment of the type 2 diabetes, metformin has been shown to exhibit antioxidant properties. As it was shown by Mahrouf et al. [15] metformin reduced intracellular ROS production in aortic endothelial cells stimulated by a short incubation with high levels of glucose. Moreover, data presented by Algire et al. [42] showed previously unrecognized inhibitory effects of metformin on ROS production and somatic cell mutation, providing a novel instrument for the reduction in cancer risk. Recently, the actual mechanism underlying the antioxidative effect of metformin was discovered. As it was shown by Hou et al. [43], metformin induced Trx expression through activation of AMPK-FOXO3 pathway, which in consequence led to reduced intracellular ROS levels. Our own data confirmed antioxidative effect of metformin as we observed decreased ROS and NO in MuASC from Met 8 group and simultaneously improved antioxidant defense originated from SOD activity. Obtained data provide a valuable information in the contexts of diabetes treatment as increased ROS production plays a key role in the onset and development of microvascular and macrovascular complications in patients with diabetes. Moreover, targeting endogenous ROS production may improve ASC metabolism and proliferative potential, making them more efficient tool in cellular therapy [44, 45].

Recently, it has been thought that metformin might improve the osteogenic differentiation potential of stromal stem cells and thus find an application in pharmacotherapy in the course of the bone healing process. Our results support this hypothesis and suggest several possible applications of metformin in regenerative medicine. Firstly, metformin can be used orally before collection of adipose tissue for isolation of ASCs for clinical application, or metformin might be used as an active agent that may be added to the culture environment to increase activity of ASCs before patient transplantation. Such supplementation might be especially useful in the elderly, as it has been previously established that osteogenic differentiation potential of ASCs declines with age [46–48]. Furthermore, the addition of proper concentrations of metformin in various types of biomaterials used for bone reconstruction may, through release into the intercellular space, increase the bone healing process.

The above applications of metformin are also supported by previously published data. It has been reported that metformin can induce MC3T3-E1 osteoblastic cell differentiation and bone matrix synthesis via AMPK activation and subsequent induction of endothelial nitric oxide synthase (eNOS) and bone morphogenetic protein-2 (BMP-2) expression [17, 18]. Metformin was also found to regulate Small Heterodimer Partner (SHP) in MC3T3-E1 cells, an orphan nuclear receptor that stimulates osteoblast differentiation and affects osteoblastic bone formation by interacting with the transcription factor Runx2 [49].

In our study, we found that 8 weeks of metformin administration in healthy mice resulted in an elevated osteogenic differentiation potential of ASCs, with simultaneous inhibition of their adipogenic differentiation ability. The maturation of ASCs into osteoblasts is pivotal for fracture healing and the osseointegration of bone-anchored implants, as well as the general bone turnover process. The osteogenic differentiation is regulated by a number of key factors and signaling pathways. Commonly used as markers, at both gene and protein levels, are BMP-2, OPN, and OCL. Here we found, on both levels, elevated expression of BMP-2, OPN, and OCL in cells derived from the Met 8 group. Interestingly, on the last day of experiment, we observed that extracellular forms of those proteins were upregulated in the Met 8, which indicates much robust osteogenic differentiation process in that group. As the mRNA levels are usually analyzed during the early phases of osteogenic differentiation, we observed only slight differences in BMP-2, OPN, and OCL expression between investigated groups. In turn, the development of large, mineralized osteonodules was also observed in Met 8 group. As such, our findings support the concept that stromal stem cells, under the systemic influence of metformin, might have wide application for use in bone healing procedures.

Our results stand in good agreement with the work by Shah et al. [50] and Zhen et al. [51], which showed that the proosteogenic action of metformin was possibly via stimulation of Runx2 and production of IGF-1. On the other hand, Wu et al. [52] demonstrated no effect of metformin on osteogenic differentiation potential of BMSCs. Kasai et al. [53], using MC3T3-E1 cells and primary osteoblasts, observed no matrix mineralization after metformin supplementation. However, both of the above mentioned studies were performed on an* in vitro* model using different doses of metformin. Along with the elevated osteogenic differentiation potential, we observed inhibition of the adipogenic differentiation ability of the ASCs isolated from both of the metformin groups. In both of these groups, the cells were characterized by a reduced proliferation activity. Furthermore, the cells did not develop the typical morphological picture for adipogenic differentiation. These results correlate with the findings of Nicpoń et al. [37], who also observed an inhibitory effect of metformin on adipogenic differentiation potential.

Besides finding that prolonged administration of metformin has a proosteogenic action, we also found that it has an impact on cortical bone density, as well as on medullary cavity volume. The combined investigation of *μ*CT and energy dispersive X-ray spectroscopy (EDX) revealed a strong correlation between calcium/phosphorus level and trabecular bone density. Our data is in agreement with the findings of Sedlinsky et al. [25], who observed that oral metformin application improves bone lesion regeneration in both control and diabetic rats. Moreover, the authors demonstrated that metformin administration enhanced the expression of osteoblast-specific transcription factor Runx2/Cbfa1 and activated AMPK in a time-dependent manner [25]. However, Jeyabalan et al. [54] demonstrated no significant differences in cortical and trabecular bone architecture in metformin-treated rodents (rat and mice after ovariectomy) compared to controls. Furthermore, the authors showed that metformin had no effect on bone resorption but reduced the bone formation rate in trabecular bone. Future studies should further assess the* in vivo* effects of metformin on bone.

## 5. Conclusions

Our results demonstrate that orally administrated metformin in healthy mice enhances the osteogenic differentiation potential of ASCs, which results in an elevated expression and secretion of BMP-2, OCL, and OPN. Additionally, we observed that metformin supplementation significantly reduced oxidative stress in MuASC and simultaneously increased SOD activity. Moreover, the development of highly mineralized osteonodules* in vitro* is enhanced by systemic metformin administration. Finally, we found that 8-week metformin supplementation leads to increased tibial bone density in mice. Our results shine a promising light on the potential application of metformin as an auxiliary strategy in healthy individuals to enhance ASCs obtained for clinical application.

## Figures and Tables

**Table 1:** Sequences of primers used in qPCR. OCN: Osteocalcin; BMP-2: Bone morphogenetic protein-2; ADPQ: Adiponectin, B2M: beta 2 microglobulin.

Gene	Primer	Sequence 5′-3′	Amplicon length (bp)	Accesion no.
OCN	F:	GGTGCAGACCTAGCAGACACCA	100	NM_001032298.2
	R:	CGCTGGGCTTGGCATCTGTAA		

BMP-2	F:	CTACAGGGAGAACACCCGGA	280	NM_007553.3
	R:	GGGGAAGCAGCAACACTAGAA		

ADPQ	F:	CCTGGAGAGAAGGGAGAGAAA	209	NM_009605.4
	R:	CGAATGGGTACATTGGGAAC		

B2M	F:	CATACGCCTGCAGAGTTAAGCA	73	NM_009735.3
	R:	GATCACATGTCTCGATCCCAGTAG		

**Table 2:** Clinical characteristics of C57BL/6 mice.

Parameters	Control group (*n* = 9)	Metformin 8 weeks (*n* = 9)
Body weight (g)	19.4 ± 0.55	22.26 ± 1.1
Fasting blood glucose (mmol/L)	13.6 ± 0.1	16 ± 0.08
Fasting serum triglyceride (mmol/L)	0.613 ± 0.018	1.25 ± 0.01
Fasting serum cholesterol (mmol/L)	1.34 ± 0.01	1.84 ± 0.02
