# Corrigendum to “Metformin Decreases Reactive Oxygen Species, Enhances Osteogenic Properties of Adipose-Derived Multipotent Mesenchymal Stem Cells *In Vitro*, and Increases Bone Density *In Vivo*”

**DOI:** 10.1155/2017/5923818

**Published:** 2017-08-20

**Authors:** Krzysztof Marycz, Krzysztof A. Tomaszewski, Katarzyna Kornicka, Brandon Michael Henry, Sebastian Wroński, Jacek Tarasiuk, Monika Maredziak

**Affiliations:** ^1^Electron Microscopy Laboratory, Wroclaw University of Environmental and Life Sciences, 5b Kozuchowska Street, 51-631 Wroclaw, Poland; ^2^Wroclaw Research Centre EIT+, 147 Stablowicka Street, 54-066 Wroclaw, Poland; ^3^Department of Anatomy, Jagiellonian University Medical College, 12 Kopernika Street, 31-034 Krakow, Poland; ^4^Faculty of Physics and Applied Computer Science, AGH University of Science and Technology, 30 Mickiewicza Street, 30059 Krakow, Poland; ^5^Department of Animal Physiology and Biostructure, Faculty of Veterinary Medicine, Wroclaw University of Environmental and Life Sciences, 31 Norwida Street, 50-375 Wroclaw, Poland

In the article titled “Metformin Decreases Reactive Oxygen Species, Enhances Osteogenic Properties of Adipose-Derived Multipotent Mesenchymal Stem Cells *In Vitro*, and Increases Bone Density *In Vivo*” [1], an acknowledgment should be added as follows:

## References

[1] Marycz K., Tomaszewski K. A., Kornicka K. (2016). Metformin Decreases Reactive Oxygen Species, Enhances Osteogenic Properties of Adipose-Derived Multipotent Mesenchymal Stem Cells *In Vitro*, and Increases Bone Density *In Vivo*. *Oxidative Medicine and Cellular Longevity*.

